# A comprehensive review of the anticancer effects of decursin

**DOI:** 10.3389/fphar.2024.1303412

**Published:** 2024-02-20

**Authors:** Yueming Chu, Qiang Yuan, Hangyu Jiang, Liang Wu, Yutao Xie, Xiaofen Zhang, Lin Li

**Affiliations:** ^1^ Department of Pharmacy, The Second Clinical Medical College of North Sichuan Medical College, Nanchong, China; ^2^ School of Pharmacy, North Sichuan Medical College, Nanchong, China; ^3^ Institute of Tissue Engineering and Stem Cells, The Second Clinical Medical College of North Sichuan Medical College, Nanchong, China; ^4^ Nanchong Key Laboratory of Individualized Drug Therapy, Nanchong, China; ^5^ Key Laboratory for Biorheological Science and Technology of Ministry of Education, Bioengineering College of Chongqing University, Chongqing, China

**Keywords:** decursin, sources, derivatives, anticancer activity, mechanisms, safety

## Abstract

Cancer is a globally complex disease with a plethora of genetic, physiological, metabolic, and environmental variations. With the increasing resistance to current anticancer drugs, efforts have been made to develop effective cancer treatments. Currently, natural products are considered promising cancer therapeutic agents due to their potent anticancer activity and low intrinsic toxicity. Decursin, a coumarin analog mainly derived from the roots of the medicinal plant *Angelica sinensis*, has a wide range of biological activities, including anti-inflammatory, antioxidant, neuroprotective, and especially anticancer activities. Existing studies indicate that decursin affects cell proliferation, apoptosis, autophagy, angiogenesis, and metastasis. It also indirectly affects the immune microenvironment and can act as a potential anticancer agent. Decursin can exert synergistic antitumor effects when used in combination with a number of common clinical anticancer drugs, enhancing chemotherapy sensitivity and reversing drug resistance in cancer cells, suggesting that decursin is a good drug combination. Second, decursin is also a promising lead compound, and compounds modifying its structure and formulation form also have good anticancer effects. In addition, decursin is not only a key ingredient in several natural herbs and dietary supplements but is also available through a biosynthetic pathway, with anticancer properties and a high degree of safety in cells, animals, and humans. Thus, it is evident that decursin is a promising natural compound, and its great potential for cancer prevention and treatment needs to be studied and explored in greater depth to support its move from the laboratory to the clinic.

## 1 Introduction

To date, a large number of nations around the globe list cancer as a main cause of death. The 2020 study reports that there are currently over 19.3 million new cases of cancer, in addition to approximately 10 million cancer deaths (Sung et al., 2021). According to recent research reports, more than 1.9 million new cancer cases are expected to occur in the United States in 2023, with approximately 609,820 people dying from cancer (Siegel et al., 2023). Cancer is a diverse and mechanistically complex disease with a plethora of underlying genetic and epigenetic factors (Han et al., 2021). It is characterized by the induction of unlimited cell proliferation and epigenetic alterations through the dysfunction of many important genes encoding key proteins (e.g., growth factors, transcription factors, anti-apoptotic proteins, and tumor suppressors) (Tarver, 2012; Millimouno et al., 2014). Currently, surgery, radiation, and chemotherapy are the main treatments for cancer. However, these therapies have serious toxic side effects on healthy human tissues and can also lead to lower success rates of standard treatment regimens, metastasis of cancer cells, excessive recurrence rates, and serious side effects (Roth et al., 2000; Liang et al., 2010; Are et al., 2018). Therefore, the search for new and effective antitumor agents is of importance. In recent years, the natural bioactive ingredients of plant origin, due to their regulation of selective molecular targets, have enormous capacity and potential for different types of cancer (Shehzad et al., 2018). Therefore, the application of traditional natural medicine monomer components for cancer treatment and prevention has received much attention.

Decursin (C_19_H_20_O_5_, from PubChem, IUPAC Name: [(3S)-2,2-dimethyl-8-oxo-3,4-dihydropyrano[3,2-g]chromen-3-yl] 3-methylbut-2-enoate), a pyranocoumarin compound extracted from the dried roots of *Angelica gigas* belonging to the Umbelliferae family, has been employed in traditional folk medicine as a tonic and a remedy for anemia (Choi et al., 2012). *Angelica gigas Nakai* (AGN) is also marketed internationally as a functional food for healthcare (Ahn et al., 2008). Decursin has been found to have a variety of therapeutic effects in increasing numbers of studies, such as anti-cancer, anti-inflammatory, anti-angiogenesis, obesity, antibacterial, and other pharmacological effects (Shehzad et al., 2018). In addition, the derivatives with bioactivity are gradually developing, suggesting that decursin can be developed as a promising lead compound. Notably, decursin has anti-cancer potential against a variety of cancers. In addition, studies have shown that chemoprevention of natural compounds through dietary regulation is a promising and cost-effective way to reduce cancer risk (George et al., 2017). Therefore, the bioactivity of plant extracts is promising and can be developed as complementary therapeutic agents or dietary supplements.

**FIGURE 1:**
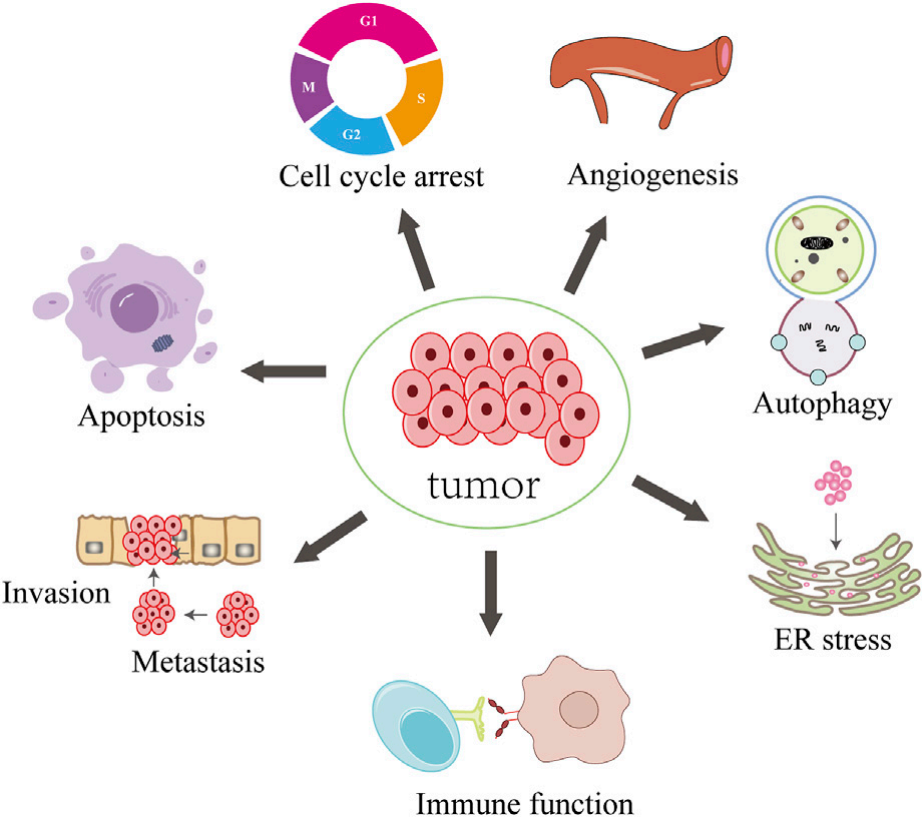
Synopsis of the mechanisms of decursin action against tumor cells.

Due to its low toxicity and anticancer potential (Ahn et al., 1996; Kim and Kim, 1999; Mahat et al., 2012; Soon-ok et al., 2012; Reddy et al., 2017), decursin has attracted increasing attention (Figure 1). Among these are influencing the immune system, limiting tumor cell growth, migration, and invasion, triggering cell death, and enhancing tumor cell sensitivity to cancer therapy. The pharmacological activity of decursin was also enhanced by modification of the active sites and the application of innovative nano-formulation methods. The anticancer potential of decursin can also be enhanced by combining it with other anticancer drugs. There are studies that have shown that the safety of dietary supplements containing decursin has been verified (Zhang et al., 2015). In this study, we first describe the natural sources, biosynthetic pathways, and active derivatives of decursin. We summarize its anticancer effects and molecular mechanisms in various cancers and update the synergistic anticancer effects of decursin with clinical drugs. It is hoped that this review will provide strong theoretical support for decursin as an anticancer drug.

## 2 Natural sources, biosynthesis of decursin, and anticancer effects

### 2.1 Natural sources of decursin and anticancer effects

**FIGURE 2:**
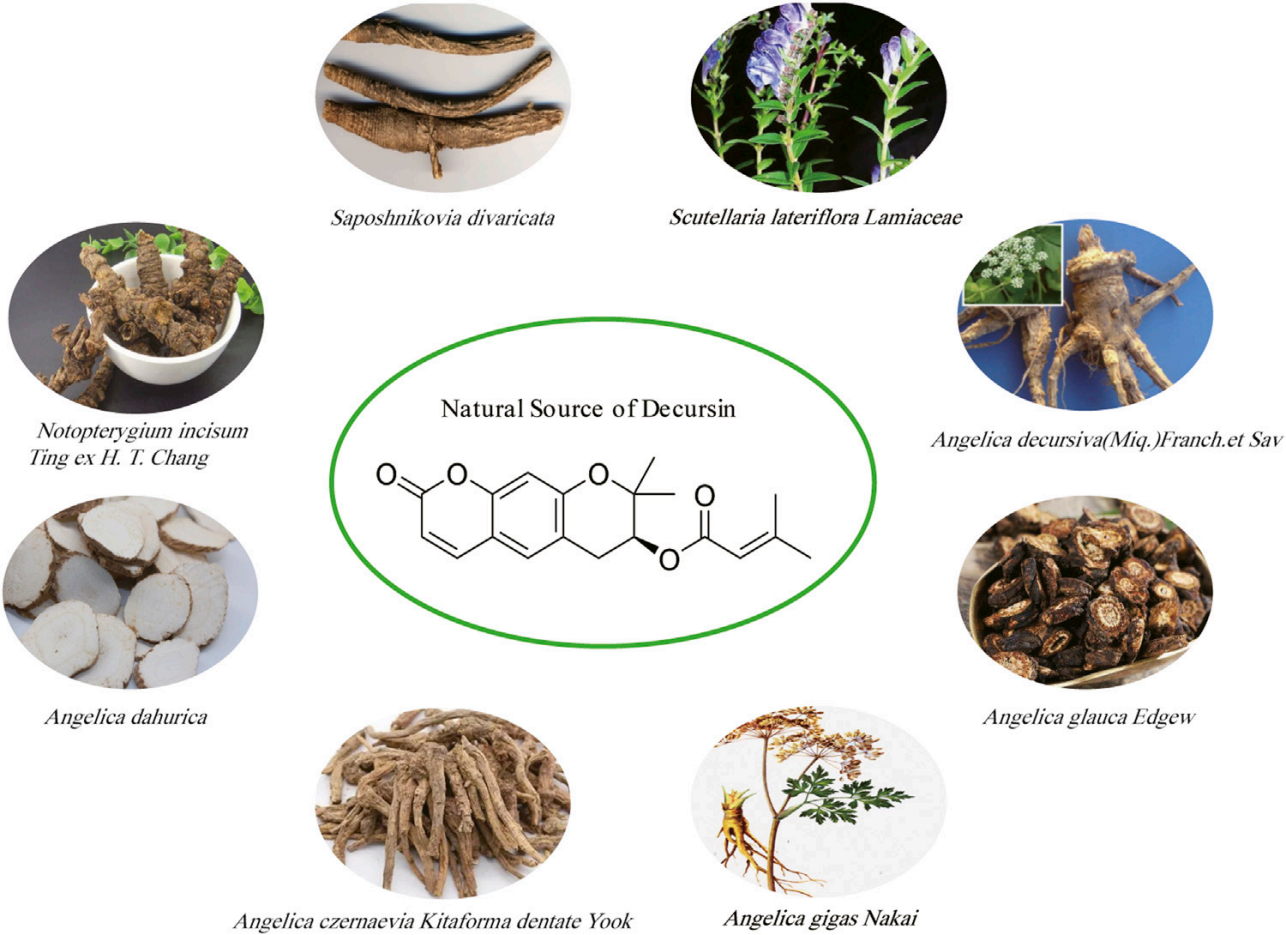
Chemical structure of decursin and its major natural sources.

Decursin is a novel pyranocoumarin compound, a monomeric component of traditional Chinese medicine, first isolated from the roots of the purple antebellum *Angelica decursiva (Franch. et Sav.)* and later from the Korean medicinal herb AGN (Hata and Sano, 1969; Ahn et al., 1996). The root of AGN is traditionally used as a folk medicine for anemia, colds, pain, and other ailments and is known to herbalists as “woman’s ginseng” (Zhang et al., 2012a). In addition, decursin was also extracted from the dried roots of the unflowered stems of *Saposhnikovia divaricata* (Zhao et al., 2010). Decursin is also extracted from the above-ground parts of *Scutellaria lateriflora Lamiaceae* (Li et al., 2009). Some other natural sources of decursin include *Notopterygium incisum Ting ex H. T. Chang*, *Angelica dahurica*, *Angelica glauca* Edgew, and *Angelica czernaevia Kitaforma* dentate Yook (Shi, 1997; Kim et al., 2006; Saeed and Sabir, 2008; Liang et al., 2018). Decursin has been extracted and isolated from a variety of herbs (Figure 2), including a study showing that in the dried roots of Korean angelica, the content has reached approximately 3% of the dried root (Ahn et al., 2008). Kweon et al. (2020) found that the ethanolic extract of AGN has good anticancer activity and confirmed decursin as the major component of AGN using an UPLC assay. Similar to AGN, the anticancer effects of *Saposhnikovia divaricata*, *Scutellaria lateriflora Lamiaceae*, *Angelica glauca* Edgew*, Angelica dahurica*, and *Notopterygium incisum Ting ex H. T. Chang* were also confirmed in several studies (Wu et al., 2010; Zheng et al., 2016; Matusiewicz et al., 2019; Park et al., 2021; Kumar et al., 2022). In addition, as shown in Table 1, we further summarized several natural sources of the active ingredient of decursin and found that decursin is not only present in AGN but also in the roots of most *Angelica genera*, such as *Angelica decursiva*, *glauca*, and *dahurica*. Second, coumarin analogs are widely found in a variety of natural plants and have good anticancer activity (Kupeli Akkol et al., 2020; Wang et al., 2021). As can be seen, decursin is a readily available and cost-effective pharmacologically active compound.

**TABLE 1:** Plant species with decursin constituents.

Plant	Plant species (family)	Medicinal part	Chemical composition	Reference
*Angelica decursiva* (Franch. et Sav.)	Umbelliferae, Angelica	Roots	Coumarins, phenolic acid, flavonoids, lignans, alkaloids, lipids, amino acids and derivatives, organic acids, nucleotides and their derivatives, terpenoids, quinones, tannins	Hata and Sano (1969), Song et al. (2022)
*Angelica gigas* Nakai	Umbelliferae, Angelica	Roots	Coumarins, volatile oils, flavonoids, polysaccharides, phthalides, nucleic acids, and organic acids	Ahn et al. (1996), He et al. (2023)
*Saposhnikovia divaricata*	Umbelliferae, Saposhnikovia	Dried roots of the unflowered stems	Coumarins, chromones, polysaccharides, organic acids, polyacetylenes, sterols, adenosine, pyrimidines, esters, alkaloid, glycerol esters, volatile oils, trace elements.	Zhao et al. (2010), Kreiner et al. (2017), Chang et al. (2022)
*Scutellaria lateriflora Lamiaceae*	Labiatae, Scutellaria	Above-ground parts	Flavonoids, volatile oils, polysaccharides, steroids, terpenoids, phenolic compounds, steroidal components, trace elements	Li et al. (2009), Huang et al. (2023)
*Notopterygium incisum* Ting ex H. T. Chang	Umbelliferae, Notopterygium	Roots and rhizomes	Coumarins, essential oils, phenoloids, amino acids, organic acids, sesquiterpenes, a polyacetylene compound, glycosides, alkaloids	Shi (1997), Zhang and Yang (2008), Azietaku et al. (2017)
*Angelica czernaevia Kitaforma*	Umbelliferae, Peucedanum	Roots	Coumarins, volatile oils	Kim et al. (2006)
*Angelica dahurica* (Fisch. ex Hoffm.) Benth. & Hook. f. ex Franch. & Sav	Umbelliferae, Angelica	Roots	Xanthotoxin, bergapten, oxypeucedanin, imperatorin, coumarin, phellopterin and isoimperatorin	Liang et al. (2018)
*Angelica glauca* Edgew	Umbelliferae, Angelica	Roots	Monoterpenes, oxygenated monoterpenes, phenylpropanoids, coumarins (decursin, decursinol angelate and bergapten), phthalides, alkaloids, carbohydrates, flavonoids, proteins, saponins, sterols, and lipids	Saeed and Sabir (2008), Kumar et al. (2022)

### 2.2 Biosynthesis pathway of decursin

**FIGURE 3:**
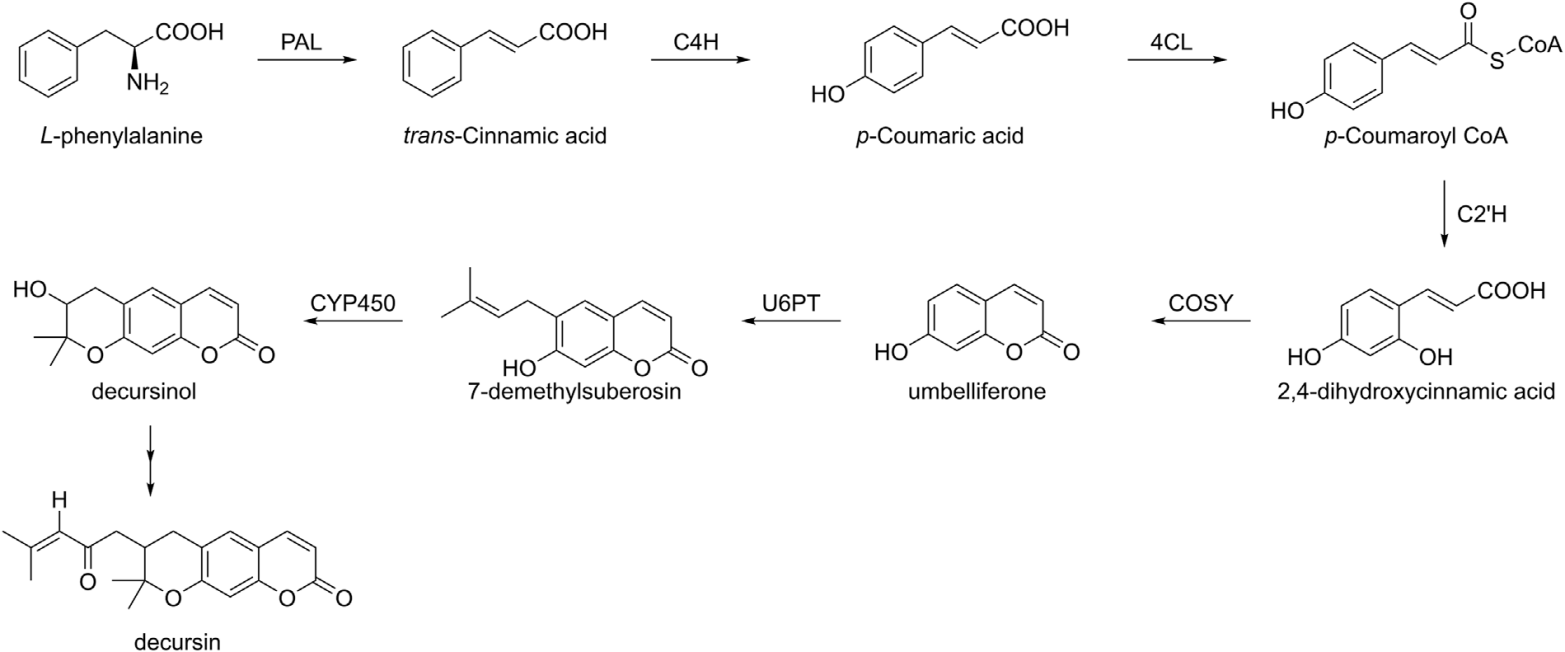
Biosynthesis pathway of decursin in plants.

Despite the high biological activity of natural products, their levels in organisms are still not sufficient to meet the requirements (Roberts et al., 2010). In addition, biosynthesis has been playing an increasingly significant role in the construction of these natural product molecules, such as podophyllotoxin and saframycin A (Yu et al., 2017; Tanifuji et al., 2018). Among them, the phenylpropanoid pathway is not only a secondary metabolic pathway in plant metabolites but also plays an important role in the biosynthesis of decursin (Figure 3). First, *L*-phenylalanine is converted to *trans*-cinnamic acid catalyzed by phenylalanine ammonia-lyase (PAL), and second, it undergoes para-hydroxylation under the action of cinnamic acid 4-hydroxylase (C4H), followed by the formation of *p*-coumaroyl CoA in the presence of 4-coumarate CoA ligase (4CL). After p-coumaroyl CoA 2′-hydroxylase (C2′H) generates 2,4-dihydroxycinnamic acid, this compound, in turn, lactonizes to form umbelliferon (Vialart et al., 2012); subsequently, umbelliferon is converted to dimethylsuberosin by the action of umbelliferone 6-prenyl transferase (U6PT). Finally, in the presence of cytochrome P-450, dimethylsuberosin is converted to decursinol and further to decursin. However, the mechanism of this last step of the biosynthetic pathway remains to be fully understood (Ji et al., 2008). Ji et al. (2008) verified the phenylpropanoid biosynthesis in AGN root cultures containing decursin using deuterium-labeled isotope data. Since PAL and C4H are important enzymes in decursin biosynthesis, transgenic hairy roots overexpressing these genes were also established (Park et al., 2010). However, this experiment does not increase decursin expression, suggesting that PAL and C4H did not affect decursin biosynthesis, whereas the effect of several other enzymes on decursin remains unknown.

## 3 Anticancer effects and molecular mechanism of decursin

### 3.1 *In vitro* anticancer effects

**FIGURE 4:**
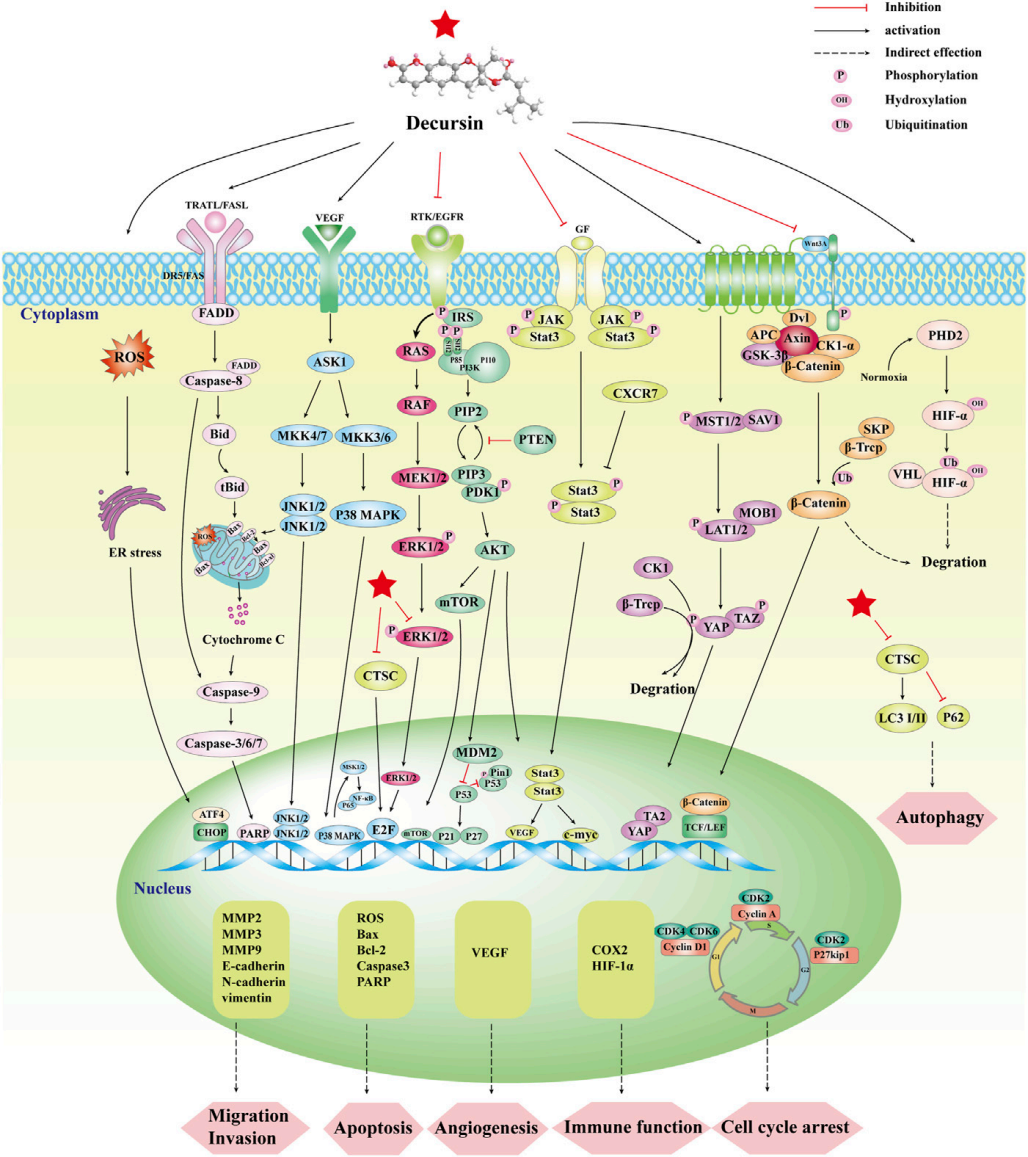
Synopsis of the mechanisms of decursin action against tumor cells.

Decursin’s anticancer activity has been clearly demonstrated in various forms of cancer, including prostate, sarcoma, breast, lung, colon, bladder, and blood cancers (Ahn et al., 2010; Kim et al., 2010d; Shehzad et al., 2018; Ge et al., 2020; Yang et al., 2023). Table 2 summarizes the anticancer effects of decursin on different cancer cells *in vitro* and the related molecular mechanisms of action. Decursin has pleiotropic effects in anticancer, including inhibition of tumor activity, cell proliferation, angiogenesis, metastasis, and invasion, induction of apoptosis and autophagy, and also affecting immune function. In this paper, we systematically summarized the anticancer effects of decursin and further analyzed the various molecular signaling pathways and molecular targets of decursin involved in tumorigenesis (Figure 4).

**TABLE 2:** Anticancer mechanisms of decursin *in vitro*

Cancer type	Cell lines	Concentration used	Treatment condition (IC_50_/treatment time)	Mechanism of action	Related protein/Pathway	Reference
Gastric cancer	SNU-216 NCI-N87	0–50 μM	50 μM(48 h) 50 μM(48 h)	Inhibits cell growth	—	Kim et al. (2021)
				Induces G0/G1 phase cell cycle arrest: CDK4/6, cyclin A, and E2F3 ↓		
				Induces autophagy: LC3-II and SQSTM1 ↑		
Gastric cancer	SNU-216 SNU-484	0–40 μM	NA	Inhibits cell growth	STAT3/c-Myc	Kim et al. (2019)
				Induces apoptosis: cleaved PARP and cleaved caspase-3 ↑; P-STAT3, c-Myc, CXCR7, and Bcl-2 ↓		
				Inhibits migration and invasion		
Bladder cancer	253J	0–100 μM	50 μM(24 h)	Inhibits cell growth	MAPK/ERK1/2	Kim et al. (2010d)
				Induces G1 phase cell cycle arrest: CDK2, CDK4, cyclin D1, cyclin E, and ERK1/2 ↓; p21 and p-ERK1/2 ↑		
				Induces apoptosis: caspase-3, cytochrome C, Bax ↑; Bcl-2 ↓		
Bladder cancer	E-J	0–100 μM		Inhibits cell growth	—	Yang et al. (2007)
Colon carcinoma	HCT116	0–100 μM	50 μM(24 h)	Inhibits cell growth	MAPK/ERK1/2	Kim et al. (2010d)
				Induces G1 phase cell cycle arrest: CDK2, CDK4, cyclin D1, cyclin E, and ERK1/2 ↓; p21 and p-ERK1/2 ↑		
				Induces apoptosis: caspase-3, cytochrome C, and Bax ↑; Bcl-2 ↓		
Colon carcinoma	HT29	0–50 μM	NA	Induces autophagy: LC3-II and SQSTM1 ↑	—	Kim et al. (2021)
Colon carcinoma	CT-26	0–20 μM	NA	Inhibits cell migration and invasion: MMP2, MMP9, P-ERK1/2, and P-JNK ↓	JNK/ERK	Son et al. (2011)
Colon carcinoma	HT29	0–90 μM	293.064 μM (24 h) 48.859 μM(48 h)	Induces apoptosis: Bax ↑ and Bcl-2 ↓	PI3K/AKT	Yang et al. (2023)
				Inhibits metastasis: E-cadherin and N-cadherin ↑; vimentin ↓		
	HCT116	0–90 μM	170.581 μM (24 h);29.461 μM (48 h)	Induces apoptosis: Bax ↑ and Bcl-2 ↓		
				Inhibits metastasis: E-cadherin and N-cadherin ↑; vimentin ↓		
Esophageal squamous carcinoma	EC109	0–80 μM	NA	Induces apoptosis: Bax, cleaved caspase-3, and ROS ↑, Bcl-2, GSH, P-JAK2, and P-STAT3 ↓	JAK2/STAT3	Ke et al. (2018)
Cervix	HeLa	0–50 μM	NA	Induces autophagy: LC3-II and SQSTM1 ↑	—	Kim et al. (2021)
Breast cancer	MCF-7	50–100 μM	NA	Inhibits cell growth Induces apoptosis: cleaved caspase-3, cleaved caspase-7, cleaved caspase-8, cleaved caspase-9, cleaved RARP, and Bax/Bcl-2 ↑	NA	Jung et al. (2019)
Breast cancer	MCF-7	0–50 μM	NA	Induces autophagy: LC3-II and SQSTM1 ↑	—	Kim et al. (2021)
Breast cancer	MCF-7	0–50 μM	NA	Inhibits cell invasion: MMP9, P-P38, and P-I-κBα ↓	MAPK/NF-κB	Kim et al. (2014b)
Breast cancer	MDA-MB-231	0–80 μM	NA	Inhibits cell viability; induces G1 phase cell cycle arrest: P53 ↑; cyclin D1 and Pin1 ↓	Pin1/P53	Kim et al. (2014a)
Breast cancer	MCF-7	0–100 μM	NA	Inhibits cell growth	—	Jiang et al. (2007)
				Induces apoptosis: cleaved PARP ↑		
				Induces G1 and G2 phase cell cycle: P-ERK1/2 ↓		
	MDA-MB-231	0–100 μM	NA	Inhibits cell growth; induces G1 and G2 phase cell cycle: p27Kip1 ↑; cyclin D1 and P21cip1 ↓	—	
Prostate cancer	LNCap	0–100 μM	NA	Induces G1 phase cell cycle arrest: cleaved caspase-3, cleaved caspase-8, cleaved -PARP, and cip1/P27 ↑; CDK2, CDK4, and cyclin D1 ↓	—	Guo et al. (2007)
Prostate cancer	PC-3	0–200 μM	NA	Inhibits cell viability; induces G1 phase cell cycle arrest: β-catenin, c-Myc, and cyclin D1 ↓	Wnt/β-catenin	Song et al. (2007)
Prostate cancer	RC-58T/h/SA#4	0–100 μM	NA	Inhibits the proliferation; induces apoptosis: cleaved caspase-3, cleaved caspase-8, cleaved caspase-9, t-Bid, Bax, cytochrome C, and AIF↑; Bid and Bcl-2 ↓	—	Choi et al. (2011)
Prostate cancer	DU145	0–100 μM		Inhibits cell growth; induces G1 phase cell cycle arrest: P107 and P130 ↑; P-ERK1/2, E2F-3, E2F-4, and E2F-5 ↓	EGFR/ERK1/2	Bhat et al. (2023)
	22Rv1	0–100 μM		Induces G1 phase cell cycle arrest: CDK4 and P27/Kip1 ↓		
Multiple myeloma cell	U266	0–160 μM	80 μM (24 h)	Inhibits cell viability	JAK/STAT3	Kim et al. (2011)
				Induces G1 phase cell cycle arrest: cyclin D1 ↓		
				Induces apoptosis: cleaved caspase-3, cleaved caspase-8, cleaved caspase-9, and cleaved RARP ↑; procaspase-3, procaspase-8, procaspase-9, Bcl-2, Bcl-xl, and survivin ↓		
				Induced angiogenesis VEGF ↓		
	ARH77	0–160 μM	80 μM (24 h)	Inhibits cell viability	—	
	MM1.S	0–160 μM	80 μM (24 h)	Inhibits cell viability	—	
Multiple myeloma cell	U266	0–160 μM	80 μM (48 h)	Inhibits cell viability	mTOR/S6K1, JAK/STAT3	Jang et al. (2013)
				Induces G1 phase cell cycle arrest: cyclin D1 ↓		
				Induces apoptosis: cleaved caspase-3, cleaved caspase-9, and cleaved RARP ↑; pro-caspase-3, pro-caspase-9, and survivin ↓		
	MM1.S	0–160 μM	80 μM (48 h)	Inhibits cell viability	—	
				Induces apoptosis: cleaved RARP ↑; pro-PARP, p-JAK2 ↓		
	RPMI8226	0–160 μM	80 μM (48 h)	Inhibits cell viability	—	
				Induces apoptosis:		
				cleaved RARP ↑; pro-PARP ↓		
Non-small cell lung cancer	A549	0–200 μM	100–200 μM (24 h)	Induces apoptosis: cleaved caspase-3, cleaved caspase-8 (44/42), cleaved caspase-8 (13), cleaved RARP, DR5, CHOP, GRP78, PERK, and ATF4 ↑	PERK/ATF4	Kim et al. (2016)
	H596					
	H1299					
Lung cancer	NCI-H460	0–50 μM	NA	Induces autophagy: LC3-II and SQSTM1↑	—	Kim et al. (2021)
Hepatocellular carcinoma cell	HepG2	0–80 μM	20 μM (48 h)	Inhibits the growth and invasion of cells	Hippo/YAP	Li et al. (2018)
				Induces G1 phase cell cycle arrest: CDK2 and CDK6 ↓		
				Induces apoptosis: cleaved caspase-3, cleaved RARP, P-YAP, P-LATS1, and βTrCP ↑; MMP, PARP, and YAP ↓		
Cervical cancer	HeLa	0–20 μM	5 μM (24 h)	Inhibits cell growth	PI3K/AKT	Zhu et al. (2021)
				Induces G1 phase cell cycle arrest: CDK2 and CDK6↓		
				Induces apoptosis: Bax, cleaved PARP, and P-AKT ↑; total caspase-3, Bcl-2, and PI3K ↓		
				Inhibits cell migration: TIMP1 ↑; MMP3 and MMP9 ↓		
Melanoma	B16F10	0–100 μM	80 μM (48 h)	Inhibits viability and proliferation	MAPK/ERK1/2	Kim et al. (2015)
				Induces apoptosis: P-P38, Bax, and pro-caspase 3 ↑; P-ERK1/2 and Bcl-2 ↓		
Glioblastoma	U87	0–200 μM	49.01 μM (24 h)	Induces G1/S phase cell cycle arrest: CDK4 and cyclin D1 ↓	—	Oh et al. (2019)
				Induces apoptosis: P-P38, P-JNK, cleaved caspase-3, cleaved caspase-7, cleaved caspase-9, and cleaved PARP ↑; Bcl-2, caspase-3, caspase-7, and caspase-9 ↓		
	C6	0–200 μM	NA	Induces apoptosis: caspase-3 ↑	—	
Oral squamous cell carcinoma	SCC-25	0–80 μM	40 μM (72 h)	Induces apoptosis: SOD, and GSH-Px ↑; MDA↓	NOX1/AKT	Wang et al. (2019)
				Inhibits proliferation and metastasis		
B-cell lymphoma	Ly1	0–90 μM	30 μM (24 h)	Induces apoptosis: cleaved PARP, caspase-3, and caspase-7 ↑; PARP, pro-caspase-3, P-AKT, P4EBP1, PS6K, P-ERK, myc, Bcl-2, Bcl-6, and MCL1 ↓	PI3K/AKT/mTOR, ERK	Kim et al. (2018)
	Ly10	0–90 μM		Induces apoptosis: myc, Bcl-2, Bcl-6, and MCL1 ↓		
	DHL6	0–90 μM		Induces apoptosis: cleaved PARP, caspase-3, caspase-7 ↑; PARP, pro-caspase-3, P-AKT, P4EBP1, PS6K, P-ERK, myc, Bcl-2, Bcl-6, and MCL1 ↓		
Ovarian cancer	NCI/ADR-RES	0–50 μg/mL	23 μg/mL (72 h)	Induces apoptosis: cleaved caspase-3, cleaved caspase-8, cleaved caspase-9, and cleaved PARP↑; Bcl-2 and P-glycoprotein↓	P-glycoprotein	Choi et al. (2016)
Pancreatic cancer	PANC-1 MIA PaCa-2	0–60 μM	40 μM (72 h)	Induces G0/G1 phase cell cycle arrest: CDK4 and cyclin D1↓	NA	Kweon et al. (2020)
				Induces apoptosis: cleaved caspase-3 and cleaved PARP↑		
				Inhibits metastasis and invasion: P-P38, MMP2, and MMP9 ↓		
Head and neck squamous cell carcinoma	SNU-1041 SNU-1076	0–100 μM	100 μM (48 h)	Inhibits cell growth	STAT3/c-Myc	Joo et al. (2022)
				Induces G0/G1 phase cell cycle arrest: cyclin A, cyclin E, CDK2, CXCR7, P-STAT3, and P-Myc ↓		
				Inhibits metastasis and invasion		
Myeloid leukemia cell	KBM-5	0–80 μM	40 μM (72 h)	Induces G1 phase cell cycle arrest; induces apoptosis: cleaved caspase-3, cleaved caspase-9, and cleaved PARP ↑; survivin, cox-2 ↓	Cox-2 and survivin	Ahn et al. (2010)

#### 3.1.1 Antiproliferative and cell cycle-blocking effects of decursin

Uncontrolled cell proliferation is a sign of cancer, and tumor cells often cause damage by directly regulating their cell cycle proteins and associated cyclin-dependent kinases (CDKs). Therefore, blocking abnormal regulation of the cell cycle is regarded as a major therapeutic method for cancer management (Sherr, 1996).

Decursin and the cell cycle arrest process are associated with the dysregulation of CDKs and the accumulation of cell cycle proteins (Kim et al., 2021; Ghafouri-Fard et al., 2022). Therefore, targeting cell cycle regulatory proteins to block cell growth provides a research direction for anticancer drug discovery. Decursin can inhibit the cell cycle at different checkpoints, blocking the G0/G1 and S phases of the cell cycle in the majority of cancers, with a small percentage of cancer cells being inhibited at the G2 phase (Jiang et al., 2007; Oh et al., 2019; Joo et al., 2022). Decursin promotes G1 phase block in 253J and HCT116 cancer cells by enhancing P21 and downregulating cyclin D1 expression through activation of the mitogen-activated protein kinase/extracellular signal-regulated kinase1/2 (MAPK/ERK1/2) signaling pathway (Kim et al., 2010d). Second, decursin induced G2 arrest at 50 μM but sparked G1 arrest at 10–20 μM in the estrogen-independent MDA-MB-231 cell line. Increased synthesis of p27Kip1 and growth-inhibitory endoplasmic reticulum (ER) protein numbers were linked to the impacts of G2 arrest (Jiang et al., 2007). However, the exact molecular mechanism by which decursin improves anticancer activity through cell cycle arrest remains unclear. Previous studies have reported that Pin1 has emerged as an essential and conserved cell cycle regulator that positively regulates cyclin D1 function in transcriptional and post-translational stabilization, causing cell cycle arrest (Liou et al., 2002). Thus, Kim et al. (2014a) are in agreement with previous studies that decursin induces G1 arrest in human breast cancer MDA-MB-231 cells mainly through Pin1/P53 signaling. Interestingly, inhibiting ERK1/2 phosphorylation prevented the G1 phase in the estrogen-dependent MCF-7 cell line (Jiang et al., 2007). Overall, decursin can target cell cycle pathways in cancer therapy.

#### 3.1.2 Induction of cancer cell apoptosis

Apoptosis is a common tumor suppression mechanism that plays an important role in cancer treatment (Morana et al., 2022). Apoptosis occurs in two typical pathways: an extrinsic pathway stimulated by death receptor activation and an intrinsic pathway activated by mitochondria-mediated endogenous stress (such as DNA damage, hypoxia, or other cellular stress), involving cysteine family proteins as well as p53 activation to initiate cell death. Fas ligand (FasL), tumor necrosis factor-alpha (TNF-α), and TNF-related apoptosis-inducing ligand (TRAIL), which function by attaching to target cell surface receptors, are the primary death ligands associated with the extrinsic route (Carneiro and El-Deiry, 2020).

Membrane proteins called death receptors, which are activated by taking in extracellular signals on the cell membrane surface, cause the external death pathway to be activated. Fas (whose ligand is FasL), the TNF receptors TNFR1 and TNFR, the TRAIL receptors death receptor 4 (DR4), and death receptor 5 (DR5) are examples of pro-apoptotic death receptors (Mohammad et al., 2015). Because of its unique capacity to kill cancer cells selectively, TRAIL is expected to be an effective anticancer therapy. Its DR5 is a transmembrane protein containing an intracellular death domain (DD), and many studies have indicated that the overexpression of DR5 leads to death receptor-induced apoptosis (Jia et al., 2012). Therefore, TRAIL resistance has been confirmed in a variety of cancers (Deng and Shah, 2020). In recent years, many natural bioactive compounds have had the potential to induce apoptosis by modulating various signaling molecules. It has been reported that decursin is able to act synergistically with TRAIL to induce apoptosis. Induction of DR5-mediated apoptosis, induction of reactive oxygen species (ROS), and selective activation of protein kinase RNA-like endoplasmic reticulum kinase (PERK)/activating transcription factor 4 (ATF4)/C/EBP homologous protein (CHOP) signaling in the ER stress pathway, which increases TRAIL sensitivity, are the primary mechanisms of action in non-small-cell lung cancer (NSCLC) cell lines (Kim et al., 2016). Consequently, decursin is an effective antitumor candidate capable of overcoming chemo-resistance in TRAIL-resistant NSCLC cells.

Members of the B-cell lymphoma-2 (Bcl-2) protein family play a major regulatory role in the endogenous pathway. Important apoptosis promoters, called BH3-only proteins, are upregulated in response to stress (such as growth factor deprivation, DNA damage, or ER stress), bind to anti-apoptotic Bcl-2 proteins with a high level of affinity, release Bcl-2-associated X protein (Bax)/Bcl-2 antagonist/killer (Bak) and form oligomers, which increase the permeability of the outer mitochondrial membrane and cause the release of apoptotic factors like cytochrome c and Smac/DIABLO from the mitochondria. This causes the cysteine cascade reaction to be activated, which causes the cleavage of hundreds of proteins and ultimately cell death (Carneiro and El-Deiry, 2020). Many natural bioactive compounds have the potential to induce internal cell death pathways by regulating various signal molecules. Studies have shown that decursin activates caspase-3 and caspase-9, members of the Caspase family proteases, by regulating circle oxymase-2 (COX-2) and survivin in leukemia KBM-5 cells and triggers the mechanism of poly ADP ribose polymerase (PARP) cutting, making human leukemia cells sensitive to cell death (Ahn et al., 2010). Further discovery that decursin induces cell death in melanoma B16F10 cells and breast cancer MCF-7 through the Bcl-2/Bax-mediated apoptosis pathway (Kim et al., 2015; Jung et al., 2019). In bladder cancer 253J and colon cancer HCT116, decursin decreases the potential of the mitochondrial membrane, makes the membranes more permeable, releases cytochrome c in the mitochondria, and activates caspase-3 to cause apoptosis (Kim et al., 2010d). However, investigations have revealed that the apoptosis-inducing factor (AIF) is released from the mitochondria and translocated to the nucleus when bladder cancer cells in RC-58T/h/SA#4 cells are in a stressful state. This causes chromosome condensation and DNA degradation, which can result in cell death (Choi et al., 2011). In multiple myeloma U266 cells, decursin regulates tumor cell viability by downregulating survivin, Bcl-2, Bcl-XL, and vascular endothelial growth factor (VEGF). It also inhibits Janus kinase 2 (JAK2) to prevent the activation of the signal transducer and activator of transcription 3 (STAT3) (Kim et al., 2011; Jang et al., 2013; Ke et al., 2018). In addition, in SNU-216, SNU-484, SNU-1041, and SNU-1076 cells of stomach cancer, decursin can also reduce c-myc expression by targeting STAT3 signals mediated by chemokine receptor 7 (CXCR7) to activate the caspase family to inhibit tumor growth and induce apoptosis (Kim et al., 2019; Joo et al., 2022). Decursin also affects the abnormal activation of the phosphoinositide 3-kinase (PI3K)/protein kinase B (Akt) signaling pathway to influence cancer growth, metabolism, and survival. It inhibits cancer cell growth and induces cysteine-dependent apoptosis in cervical cancer, B-cell lymphoma, colon cancer, oral squamous carcinoma, liver cancer, pancreatic cancer, and glioma (Kim et al., 2018; Li et al., 2018; Oh et al., 2019; Wang et al., 2019; Kweon et al., 2020; Zhu et al., 2021; Yang et al., 2023). Doxorubicin-resistant ovarian cancer cells NCI/ADR-RES induce apoptosis in the presence of decursin treatment. The main mechanism is that decursin induces apoptosis by blocking P-glycoprotein expression, activating caspase family activity, and increasing cleaved PARP levels in the presence of doxorubicin (Choi et al., 2016).

It has been established that apoptosis is a crucial intracellular process for preserving homeostasis and regulating the number of cells in the body. Decursin can promote apoptosis through multiple signaling pathways, signaling axes, or some target proteins in different cancers and is expected to be a novel anticancer agent and a potential new treatment option for multi-drug-resistant tumors.

#### 3.1.3 Inhibition of invasion and metastasis of cancer cells

Despite the nearly century-long development of anticancer drugs, the rate of 5-year survival for patients with metastatic cancer, especially distant metastases, is still dismal (Steeg, 2016). In addition, secondary tumors are clinically detectable in only mature stages, frequently after several metastases have occurred. It is well-recognized that the invasion and migration of tumor cells to nearby tissues or organs is a dynamic, multi-stage process known as tumor metastasis (Li et al., 2017).

A number of key molecules are involved in the adhesion, migration, and invasion of cancer cells. These include matrix metalloproteinases (MMPs), which are helpful for tumor invasion and migration because they breakdown the extracellular matrix. The extracellular matrix (ECM) is destroyed by cancer cells, allowing them to penetrate healthy tissues (Jablonska-Trypuc et al., 2016; Huang et al., 2021). By breaking down practically all ECM protein components, destroying the histological barrier to tumor cell invasion, and being a crucial factor in tumor invasion and metastasis, MMP plays a significant role in the development of cancer (Said et al., 2014). MMPs have consequently turned into desirable targets for oncology research and the creation of antitumor medications. In addition, epithelial cells are changed into mesenchymal cells by the complex biological process of epithelial–mesenchymal transition (EMT), which also gives them the ability to move and invade (Son and Moon, 2010; Ribatti et al., 2020). There is strong evidence that EMT can promote cell motility and invasiveness, thereby allowing cancer cells to detach from the primary mass and spread to secondary sites (Son and Moon, 2010; Ribatti et al., 2020). When EMT occurs, the expression of the epithelial marker E-cadherin is downregulated, whereas the mesenchymal marker N-cadherin is upregulated (Loh et al., 2019). A recent study demonstrated that decursin inhibited the proliferation and EMT of HT29 and HCT116 colon cancer cells by downregulating N-cadherin and vimentin protein expression and upregulating E-cadherin and PI3K/AKT signaling pathway expression, thereby inhibiting metastasis (Yang et al., 2023). Additionally, it was discovered that decursin inhibited lung metastasis and CT26 cell invasion in colon cancer cells, mostly via suppressing the expression of ERK/c-Jun N-terminal kinase (JNK) but not the p38 MAPK pathway (Son et al., 2011). The results showed that decursin strongly inhibited CT 26 metastasis formation and was as effective as celecoxib against invasion. In contrast, the p38-dependent pathway is associated with the expression and activation of MMPs in pancreatic cancer cells; thus, decursin inhibits migration and invasion of pancreatic cancer (PANC-1 and MIA PaCa-2) cell lines mainly through phosphorylation of p38 to control the expression of MMP-2 and MMP-9 (Kweon et al., 2020). In breast cancer MCF-7 cell lines, the anti-metastatic mechanism of decursin remained distinct. Decursin inhibits 12-O-tetradecanoylphorbol-13-acetate (TPA)-induced translocation of protein kinase C α (PKCα) from cytoplasmic lysate to the membrane rather than impacting PKCδ translocation, primarily through the MAPK/nuclear factor-κB (NF-κB) pathway, to affect MMP-9 production and cell invasion (Kim et al., 2014b). Among them, decursin increased cell proliferation, growth, migration, and invasion in gastric cancer by downregulating CXCR7 to affect the STAT3/c-Myc pathway (Kim et al., 2019). In addition, the specific mechanism of migration and invasion inhibition by decursin in head and neck squamous cell carcinoma (SNU-1041 and SNU-1076) and hepatocellular carcinoma (HepG2) is unclear and needs further insight to enable the natural product and its derivatives to play an important role in inhibiting the activity of MMP-2 and MMP-9 (Li et al., 2018; Joo et al., 2022).

#### 3.1.4 Suppression of the angiogenesis of cancer cells

Cancer cells require nutrients as well as oxygen to survive and multiply, and being close to blood vessels provides them access to the circulatory system. Additionally, the creation of blood vessels is required for tumor development. Therefore, inhibiting angiogenesis can inhibit tumor growth and spread, making cancer a manageable chronic condition (Son et al., 2009; Lugano et al., 2020). Pro-angiogenic factors and their associated receptors in abundance, primarily VEGF, fibroblast growth factor 2 (FGF-2), platelet-derived growth factor (PDGF), and angiopoietins, stimulate the formation of new blood vessels in tumors (Lugano et al., 2020). Among them, one of the most effective agents for promoting angiogenesis is VEGF (Ferrara et al., 2003). VEGF is produced and secreted by cancerous tumor cells and the stroma that surrounds them. It is linked to tumor progression, increased vascular density, invasiveness, metastasis, and tumor recurrence (Apte et al., 2019). Jung et al. (2009) revealed for the first time that decursin had excellent inhibitory effects on VEGF-induced vascular formation in human umbilical vein endothelial cells (HUVECs), fertilized eggs, and intramuscular animal models *in vitro*. Son et al. (2009) proceeded on to clarify that decursin had significant *in vivo* antiangiogenic effects, primarily via preventing the angiogenesis generated by vascular endothelial growth factor by reducing ERK and JNK activation in HUVECs. In vascular endothelial cell morphogenesis, MAPK signaling is a key molecular event in VEGF-induced proliferation, survival, and migration. Decursin inhibits VEGF-induced angiogenesis primarily by decreasing ERK and JNK activation. Consequently, decursin has the potential to be a novel angiogenesis inhibitor (Rousseau et al., 2000; Son et al., 2009). In a mouse model of oxygen-induced retinopathy, decursin also reduced blood retinal barrier breakdown, retinal angiogenesis, and migration of retinal endothelial cells via blocking the VEGFR-2 signaling pathway (Kim et al., 2009a). It was further shown that decursin may inhibit VEGFR-2 transcription or activation by directly regulating VEGFR-2 phosphorylation or by blocking VEGF binding to its receptor VEGFR-2, while the effect on endothelial progenitor cell (EPC) differentiation may be through the VEGF/VEGFR-2 and stromal cell-derived factor 1 (SDF-1)/CXCR4 signaling pathways, further demonstrating the good preventive potential of abscisin in the early stages of tumor formation in EPCs (Jung et al., 2012). The Lee et al. (2010) patent also demonstrates that decursin-containing compositions are effective in the treatment of VEGF-induced angiogenesis-related disorders. Thus, decursin primarily inhibits VEGFR2-mediated angiogenesis to prevent early tumor development.

#### 3.1.5 Regulation effect of decursin on immunity

The human immune system, with its own adaptive and innate immunity, is able to recognize and kill abnormally proliferating cells, thereby eliminating tumor cells or controlling tumor growth. Among other things, immune cells are able to infiltrate the tumor microenvironment (TME) to regulate tumor progression (Palazon et al., 2012). T cells, B cells, and natural killer (NK) cells are adaptive immune cells that can be activated to produce an immune response by exposure to specific antigens. Macrophages and neutrophils, which carry out the innate immune response, are a non-specific defense mechanism that will act within hours of the entry of a foreign antigen into the body (Hinshaw and Shevde, 2019; Anderson and Simon, 2020). Cancer cells can shape the microenvironment to help support tumorigenesis and evade the immune system through suppression (Arner and Rathmell, 2023). It has been suggested that this phenomenon develops as a result of crosstalk between cancer cells and proximal immune cells (Hinshaw and Shevde, 2019). Thus, immune cells are an important part of the matrix of the tumor microenvironment. Reports are suggesting that hypoxia-stabilized HIF-1α can mediate the tumor immune response by inducing the expression of the immune checkpoint programmed cell death ligand 1 (PD-L1), which leads to immunosuppression and evasion, ultimately leading to tumor growth (Noman et al., 2014; Palazon et al., 2014). In recent years, Ge et al. (2020) demonstrated for the first time that decursin reduces HIF-1 protein accumulation by promoting ubiquitination and proteasomal degradation. They also demonstrated that in the xenograft mouse tumor model, decursin increased infiltrating lymphocytes (CD3^+^), helper T cells (CD4^+^), and cytotoxic T cell (CD8^+^) accumulations while decreasing tumor expression of regulatory T cells (Foxp3) and myeloid-derived suppressor cell-mediated immunosuppressive factor (Arg1) in tissues. Therefore, decursin is a promising novel HIF-1α inhibitor that can directly inhibit the expression of PD-L1, which can help improve T-cell activation in the tumor microenvironment and inhibit the growth of tumor tissues. To confirm whether decursin may be employed as an immunotherapeutic anticancer agent, however, plenty of research is still required.

#### 3.1.6 Induction of cancer cell autophagy

Autophagy, which is a mechanism for delivering cellular material to lysosomes for degradation, is an evolutionarily conserved catabolic process that leads to a basal turnover of cellular components and provides energy and macromolecular precursors (Levy et al., 2017). It has been demonstrated that autophagy is crucial for cancer cell survival and adaptation to changes in the tumor microenvironment under diverse stress circumstances and that autophagy is crucial for maintaining cellular homeostasis as a protein/organelle quality control process (Nazio et al., 2019). Autophagy dysregulation is frequently linked to the development of cancer. The creation of membrane-bound vacuoles, or autophagosomes, in the cytoplasm as a result of nutritional deprivation, which contain cytoplasmic organelles, such as organelles and inclusions, is one characteristic of autophagy. Autophagy-related protein (ATG), which includes Beclin1 and microtubule-associated protein light chain 3 (LC3), is the primary signaling molecule in autophagy (Chifenti et al., 2013). Currently, the most widely studied ATG8 protein in mammalian cells is LC3 (Mareninova et al., 2020). The results of existing studies show that LC3-II levels are an important indicator of cellular autophagic activity (Plaza-Zabala et al., 2020). After treatment with decursin, an increase in LC3II/LC3I and Sequestosome 1 (SQSTM1) levels was observed in a time-dependent manner, consistent with the inhibitory effect of treatment with bafilomycin A, a well-known autophagy inhibitor, suggesting that autophagic fluxes are affected by decursin (Kim et al., 2021). Second, decursin-mediated inhibition of autophagic flux was observed in gastric, colon, cervical, breast, and lung cancer cells (Kim et al., 2021). The role of autophagy in tumors is complex, and the therapeutic potential of interventions that activate or inhibit autophagy is enormous (de Souza et al., 2020; Debnath et al., 2023). Therefore, there is a need to further investigate autophagy and lysosomal proteases in decursin-mediated tumorigenesis and development and fully exploit the therapeutic potential of autophagy modulators.

#### 3.1.7 Decursin in combination with chemotherapy drugs

Combination therapy is a useful method for overcoming drug resistance in all varieties of malignant diseases affecting humans. Currently, molecular targeted therapy, chemotherapy, radiotherapy, and surgical resection are the primary cancer treatments (Debela et al., 2021; Saini and Twelves, 2021). However, some tumors develop drug resistance quickly, and tumor cell resistance is a significant factor in treatment failure and a poor prognosis for oncology chemotherapeutic treatments. It has been observed that using two or more medications in combination therapy can increase effectiveness and lower the occurrence of drug resistance (Vasan et al., 2019; Casak et al., 2021). Additionally, there is mounting evidence that natural compound combination therapy may be more beneficial than monotherapy, which aims to enhance effectiveness while limiting the possibility of adverse medication reactions (Zacchino et al., 2017). Decursin is a novel inhibitor of STAT3 activation, and when combined with bortezomib, it increases cytotoxicity and induces cell death in human multiple myeloma cells (Kim et al., 2011). Decursin, in combination with doxorubicin, enhances mitochondrial apoptosis in multiple myeloma cells by decreasing mitochondrial membrane potential through the mechanistic target of the rapamycin (mTOR)/STAT3 signaling pathway, decreasing cyclin D1 and survivin expression, and thereby inhibiting the negative regulation of the upstream complex phosphatase (PTP) of the STAT pathway (Jang et al., 2013). The anticancer activity of doxorubicin has reportedly been shown to inhibit DNA polymerase and topoisomerase II (Zunina et al., 1975; Tewey et al., 1984). Therefore, it may be inferred that the suppression of DNA polymerase or topoisomerase II activity may contribute to the synergistic anticancer action of decursin and doxorubicin. However, more experiments are still needed in the near future to test this hypothesis (Jang et al., 2013). Interestingly, decreased P-glycoprotein expression by decursin also increased the chemosensitivity of doxorubicin. By reducing P-glycoprotein expression, decursin and doxorubicin together cause apoptotic cell death in doxorubicin-resistant ovarian cancer cells. Although NF-κB can regulate the gene expression of P-glycoprotein, the specific mechanism by which it affects decursin synergistically with doxorubicin needs to be further explored (Choi et al., 2016). Decursin substantially recovered Cu/Zn superoxide dismutase (SOD), catalase, and glutathione peroxidase activity in cisplatin-treated human renal epithelial cells, according to Kim et al. (2010b), whereas the known antioxidant N-acetyl-L-cysteine (NAC) did not restore levels of antioxidant enzymes inhibited by cisplatin treatment. Combination treatment with decursin and cisplatin significantly protected human primary renal epithelial cells (HRCs) from cisplatin-induced cytotoxicity and apoptosis. Therefore, for multi-drug-resistant (MDR) tumor, decursin may be a viable new treatment option. However, although the combination of decursin with chemotherapeutic agents can increase efficacy and has shown some safety in *in vivo* trials in animals, its results *in vivo* need to be further studied in large samples.

### 3.2 *In vivo* antitumor activity

The *in vivo* antitumor activity of decursin was significant in different types of tumors (Table 3). Among them, BALB/c nude mice, C57BL/6J mice, pathogen-free male ICR mice, and Eμ-myc transgenic mice are commonly used models for *in vivo* anticancer studies of decursin . Tumor models are established by subcutaneous, intraperitoneal, or oral implantation of mouse cancer cells or xenografts to form tumors (Lee et al., 2003; Jung et al., 2012; Kim et al., 2018; Kim et al., 2021). Decursin was reported to significantly suppress tumor size in gastric cancer xenograft mice by disrupting autophagy, inhibiting cathepsin C (CTSC), and downregulating CXCR7 (Kim et al., 2019; Kim et al., 2021). Decursin suppresses hepatocellular carcinoma growth in nude mice via the Hippo/YAP signaling system (Li et al., 2018). Similarly, decursin inhibited the production of cervical cancer cells in mice *in vivo* (Zhu et al., 2021). When compared to the effects of phosphate-buffered saline (PBS) therapy, decursin significantly decreased tumor weight and growth. Tumor tissue homogenate analysis of the B16F10 tumor revealed that the decursin-treated group was able to promote tumor apoptosis *in vivo* by reducing procaspase-3 protein expression *in vivo* (Kim et al., 2015). Furthermore, decursin suppressed the proliferation and invasion of CT-26 colon cancer cells along with lung metastases in mice by decreasing MMP-9 production via the ERK/JNK signaling pathway (Son et al., 2011). Decursin administration totally restored the normal appearance of the spleen in Eμ-myc transgenic mice, which was closely associated with a massive decrease in Myc expression, showing that decursin could be effective in the treatment of Myc-driven B-cell lymphoma (Kim et al., 2018). Decursin was also found to inhibit sarcoma size *in vivo* in pathogen-free male ICR mice, tumor growth in prostate cancer in C57BL/6 TRAMP mice, and angiogenesis in C57BL/6J mice in liver cancer (Lee et al., 2003; Jung et al., 2012; Tang et al., 2015). Finally, in the esophageal squamous carcinoma, decursin increased the expression of the pro-apoptotic protein Bax and the cleavage activation of caspase-3 through the JAK2/STAT3 pathway and inhibited the expression of anti-apoptotic proteins, thus promoting apoptosis in tumor cells (Ke et al., 2018). *In vivo* anticancer studies of decursin show the potential of decursin to treat cancer. Unfortunately, only *in vivo* rodent studies are insufficient to demonstrate that decursin may be used clinically as an anticancer drug. As a result, the anticancer potential of decursin has to be investigated further *in vivo*.

**TABLE 3:** *In vivo* anticancer properties of decursin.

Cancer type	Animal model and site of tumor xenograft	Cell lines	Dose, duration, and the route of administration	Observation and mechanism of action	Efficacy on tumor inhibition	Reference
Gastric cancer	BALB/c-nude mice	NCI-N87	50 μM, twice per week, subcutaneous	Inhibits tumor growth: CTSC, E2F3, and Bcl-2 ↓	In contrast to control mice, decursin-treated mice showed retarded tumor growth; body weight was not affected by the treatment	Kim et al. (2021)
Gastric cancer	Four-old female BALB/c nude mice	SNU-484-CXCR7	40 μM, SNU484-CXCR7 cells pretreated with 40 μM decursin for 24 h subcutaneous	Inhibits tumor growth: CXCR7↓	Decursin reduced cell survival and growth through the downregulation of CXCR7 *in vivo*	Kim et al. (2019)
Hepatocellular carcinoma cell	Six-week-old male BALB/c nude mice	HepG2	30 mg/kg, every 2 days, 3 weeks of treatment, intraperitoneally	Inhibits tumor growth: Ki-67 tissue level↓	Reduces the *in vivo* tumor growth	Li et al. (2018)
Cervical cancer	BALB/c nude mice	HeLa	30 mg/kg, every 2 days, 3 weeks of treatment, subcutaneous	NA	Decreases the tumor formation	Zhu et al. (2021)
Melanoma	Male C57BL/6J mice	B16F10	10 mg/kg, alternate days for 14–20 days with a total volume of 0.1 mL, subcutaneous	Inhibits tumor growth: pro-caspase-3↓	Inhibits tumor growth	Kim et al. (2015)
Colon carcinoma	Six-week-old male BALB/c nude mice	CT-26	10 mg/kg, once a day for 2 weeks, oral gavage	NA	Inhibits proliferation and invasion of CT-26 colon carcinoma cells, as well as the lung metastasis of CT-26 cells in mice	Son et al. (2011)
B-cell lymphoma	Eμ-myc transgenic mice	—	10 mg/kg, 4 weeks, /	Attenuates lymphogenesis: Myc↓	Decursin completely restored the normal appearance of the spleen	Kim et al. (2018)
Sarcoma	Pathogen-free male ICR mice	Sarcoma 180	50 and 100 mg/kg, nine consecutive days, intraperitoneally	NA	Inhibition of tumor volumes	Lee et al. (2003)
Prostate carcinoma	Male C57BL/6J TRAMP mice	—	3 mg/mouse, 5 days per week, 16 weeks, oral gavage	Inhibits tumor growth: Ki67	Inhibits tumor growth	Tang et al. (2015)
Lung cancer	C57BL/6J mice	LLC	4 mg/kg, intraperitoneally	NA	Inhibits vasculogenesis	Jung et al. (2012)
Esophageal squamous carcinoma	BALB/c nude mice	EC109	4 mg/kg, every 2 days, 3 weeks of treatment, subcutaneous	Induces tumor growth: Bax, cleaved caspase-3, ROS ↑, Bcl-2, GSH, P-JAK2, and P-STAT3 ↓	Inhibits tumor growth	Ke et al. (2018)

## 4 Active derivatives of decursin and antitumor effects

Currently, there are many natural compounds that have very low potential for direct use as phytotherapeutic agents due to disadvantages such as poor water solubility and high toxicity (Atanasov et al., 2021). The most common strategy is to create analogs by modifying the structure (Maier, 2015). Although the antiproliferative effects of decursin are well known, they are not sufficient to be approved for clinical use.

**FIGURE 5:**
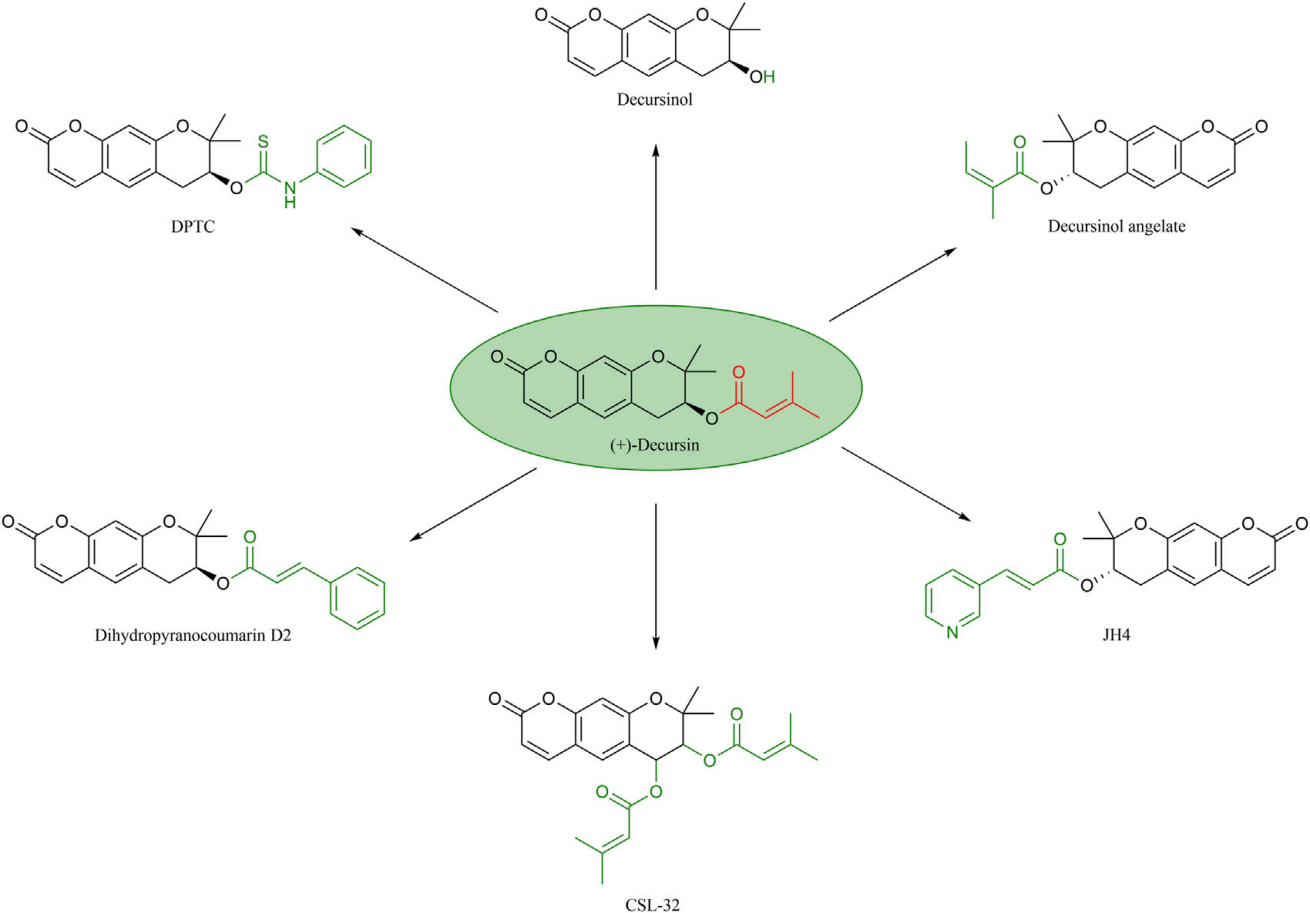
Structure–activity relationship of decursin.

Decursin is a biologically active natural compound that, due to its hydrophobic nature, is extracted using methods such as ethanol or supercritical carbon dioxide fluids (Zhang et al., 2012a). In order to further explain the effect of different structures on the antitumor activity of decursin, the corresponding structure–activity relationships were systematically summarized, and the structures of decursin and its analogs are shown in Figure 5. It was found that the C-7 position side chain of decursin was replaced by 1-phenyl acrylate to generate decursinol angelate, which showed reduced spatial site resistance and significantly increased anticancer activity. Decursinol angelate was able to significantly inhibit the invasion of the fibrosarcoma cell line HT1080 and the breast cancer cell line MDA-MB-231, as well as inhibit the increase of B16F10 melanoma cells (Kim et al., 2010c; Chang et al., 2021). Second, decursinol is not only the active derivative of decursin but also its metabolite (Yim et al., 2005; Lee et al., 2009). The side chain of decursin (CH3)2-C = CH-COO is replaced by -OH, but decursinol, which has the same coumarin ring system, is not as anticancer as decursin, suggesting that this isoprenoid structure may be important for the anticancer effect (Kim et al., 2005; Yim et al., 2005; Song et al., 2007). Interestingly, the Son team further showed that oral administration of decursinol at 10 mg/kg resulted in a significantly better reduction of lung tumor nodules in mice than decursin at the same dose (Son et al., 2011). It can be seen that although decursinol lacking a side chain is not as effective as decursin against cancer, it shows superior anti-metastatic ability *in vivo* than decursin. Zhang et al. (2012b) synthesized a decursin derivative, decursinol phenylthiocarbamate (DPTC), whose phenylthiocarbamate group modification of the side chain not only retained the anti-AR activity *in vivo* and *in vitro* but also exhibited greater hydrolysis resistance than decursin. Consequently, DPTC would not be converted into DOH *in vivo*. At the same time, its anticancer efficacy was somewhat diminished due to its low solubility and low bioavailability. Therefore, the substituent group at the C-7 position of decursin is a determinant of AR antagonist activity. In addition, the introduction of (CH3) 2-C = CH-COO at the C-7 and C-8 positions of decursin produces a more active decursin derivative, CSL-32, which inhibits the expression of pro-inflammatory mediators that invade or migrate through the extracellular matrix and human fibrosarcoma cells in the presence of TNF (Lee et al., 2012). This suggests that the increased lipid solubility of decursin at the C-8 position facilitates more favorable binding to amino acid residues of proteins, resulting in a more stable binding and a greater impact on anticancer effects. Lee et al. (2019) also introduced 3-(3-pyridyl) acrylic acid at the C-7 position and synthesized the decursin derivative JH-4, which can effectively block the progerin–lamin A/C combination and treat a variety of severe vascular inflammatory diseases. Therefore, its anticancer potential warrants further investigation. There are recently disclosed patents indicating that dihydropyranocoumarin D2 is a pharmaceutical composition with anticancer potential capable of combining with Bruton’s tyrosine kinase inhibitor (Jeon et al., 2022). In addition, it has also been shown that the derivative dihydropyranocoumarin D2 is a promising lead compound that can effectively regulate tyrosinase and thus inhibit melanogenesis due to its strong potential (Kim et al., 2010a). Furthermore, it has been shown that decursin and its derivative, decursinol angelate, inhibit melanin formation in B16 murine melanoma cells (Zhang et al., 2012b). Therefore, there is a need to further investigate whether dihydropyranocoumarin D2 has considerable potential to be effective against melanoma.

In conclusion, decursin is an important prototype lead compound, and the C-7 position of decursin is an important active site that can be altered in terms of lipid solubility and bioavailability through the introduction of various groups, with a view to designing more decursin derivatives with higher stability and broader application prospects.

## 5 Targeting the drug delivery system of decursin and its anticancer effects

Decursin has limited solubility and bioavailability, which creates difficulties for drug delivery systems and targeted drug release. In recent years, nanoparticles (NPs) have been repeatedly reported to play an important role in modern medicine. The rapid growth of nanotechnology toward the development of nanomedicines holds great promise for the improvement of cancer therapeutics (Aghebati-Maleki et al., 2020). Due to the poor water solubility of decursin, in order to further improve it, Lee et al. (2017) prepared the first nanocomposites (NCs) of AGN ethanol extract (decursin as the main component) by using a modified nanocrystal preparation method and further found that the group of AGN NCs was capable of promoting apoptosis generation. The anticancer activity in breast cancer was significantly higher than that of the AGN EtOH extract group and had no effect on normal cells, suggesting that AGN nanoformulations may have tumor-selective killing efficacy. This was further validated, and polydopamine (PD)-coated NCs were investigated. It was found that both PD-AGN NCs and AGN NCs had better activity than the AGN EtOH extract. The PD layer of PD-AGN NCs appeared to have better cell adhesion in MDA-MB-231 cells. In addition, they contribute to apoptotic and antiproliferative efficacy (Nam et al., 2018b). Second, Nam et al. (2018a) further showed that AGN NPs act through endocytosis into the cytoplasm rather than the nucleus. This shows that nanoformulations improve the aqueous solubility, bioavailability, and targeting of desmoglein for better application in tumor therapy.

## 6 Safety or low toxicity of decursin in cells, animals, and humans

Decursin is crucial for the treatment of a variety of illnesses, including cancer. In recent years, it has been shown that *in vitro* experiments based on two-dimensional cultures do not accurately reflect the environment of the patient (Edwards et al., 2015). As a result, it is critical to assess its toxicity and safety levels before using it in cancer treatment. In recent years, it has been discovered that not all PKC activators are tumor promoters (Ruan and Zhu, 2012). Interestingly, Ahn et al. (1996) demonstrated *in vitro* for the first time that decursin was more effective in blocking rapidly growing cancer cells than slowly growing normal fibroblasts and that the cytotoxic activity of decursin may be related to the mechanism of PKC activation, but further exploration is still needed *in vitro*. Kim et al. (2021) established a spheroid 3D culture, a patient-derived organoid model, and a rodent xenograft tumor model to show that 50 μM of dexmethylphenidate is toxic to cancer cells but has a good safety profile and has no effect on body weight in mice. Similarly, decursin showed no toxic effects on normal human cell lines (Ahn et al., 2010; Kim et al., 2011; Kweon et al., 2020). By modulating COX-2 and survivin, decursin induces an apoptotic process that is safe for human peripheral blood lymphocytes but detrimental to leukemia cells (Ahn et al., 2010). Decursin, an agent used to treat variable retinopathy caused by a breakdown of the blood–retinal barrier (BRB), did not produce retinal toxicity at concentrations up to 50 mol/L, which is five times the therapeutically effective value. This implies that decursin can be used to treat this problem without putting the retina or healthy retinal vessels at risk (Kim et al., 2009b). In addition to investigating the safety of decursin in cell lines and rodents, a related clinical trial has also investigated the pharmacokinetics of a single oral dose of decursin and the decursinol angelate-enriched dietary supplement Cogni-Q. In humans, a total of 20 healthy subjects, each taking 119 mg of decursin and 77 mg of decursinol angelate, were enrolled in the pharmacokinetic study. The results of the study provide credibility to the safety data using rodent models (Zhang et al., 2015). The above studies facilitate the clinical translation of decursin against cancer and other diseases and explore relevant active molecular targets. However, further studies are still needed to assess the genotoxicity and reproductive toxicity of decursin, among others, in order to support anticancer studies of decursin.

## 7 Conclusion and future perspectives

In recent years, natural medicine therapies have shown great potential in the treatment of cancer. Decursin is a natural compound that, through abnormal signaling and epigenetics, inhibits the growth, invasion, migration, apoptosis, autophagy, and immunity of cancer cells. Although previous studies have shown that decursin has good anticancer effects and a certain degree of safety, as the current studies on its anticancer effects are still in the preclinical stage, only one of its safety studies has been marketed abroad as a dietary supplement. This shows that, due to the lack of research in the clinical stage, decursin needs to be explored comprehensively before it can be established as an anticancer agent. As a result, we propose some ideas that we hope will serve as a direction for further research. First, due to the limited solubility and bioavailability of ecdysteroids, there is a need to structurally modify their active side chain at the C-7 position and to investigate nanoformulations and other types of novel targeted delivery systems to enhance their anticancer potential. Second, decursin is able to inhibit tumor growth by targeting CXCR7, but limited research has been conducted, and the mechanism is unknown. Therefore, the potential of decursin as a CXCR7-targeting agent warrants further research. Third, chemotherapy resistance is still the key factor contributing to poor survival and prognosis in tumor patients. Whether decursin can be used as a combination of chemotherapeutic drugs to reduce chemotherapy drug resistance still needs further in-depth validation. Fourth, so as to maximize the anticancer potential of decursin, additional studies are required on the main mechanisms underlying particular forms of death (autophagy, ferroptosis, pyroptosis, and cuproptosis), which have not been conducted. Fifth, the safety of decursin has been shown in rodent *in vivo* studies in rodents but remains insufficient in the clinical trial part. Finally, decursin is being used as the primary active element in the nutraceutical Cogni-Q. As a result, more research is needed to discover whether decursin may be utilized as a medical herbal monomer and whether it can be employed in the prevention and treatment of cancer.

## Figures and Tables

**Table:** 

**MMPs**	Matrix metalloproteinases
**EMT**	Epithelial–mesenchymal transition
**AGN**	Angelica gigas Nakai
**NCs**	Nanocomposites
**DPTC**	Decursinol phenylthiocarbamate
**AR**	Androgen and the androgen receptor
**DOH**	Decursinol
**DA**	Decursinol angelate
**CSL-32**	A derivative of decursin
**MAPK**	Mitogen-activated protein kinases
**NF-κB**	Nuclear factor-κB
**HGPS**	Hutchinson–Gilford progeria syndrome
**ECM**	Extracellular matrix
**TNFα**	Tumor necrosis factor α
**PI3K**	Phosphoinositide 3-kinase
**Akt**	Protein kinase B
**LC3**	Microtubule-associated protein light chain 3
**PK**	Pharmacokinetic
**CDKs**	Cyclin-dependent kinases
**MAPKs**	Mitogen-activated protein kinases
**ERKs**	Extracellular signal-regulated kinases
**TNF-α**	Tumor necrosis factor-alpha
**FasL**	Fas ligand
**TRAIL**	Tumor necrosis factor-related apoptosis-inducing ligand
**DR**	Death receptor
**DD**	Death domain
**ROS**	Reactive oxygen species
**ATF4**	Activating transcription factor 4
**CHOP**	C/EBP homologous protein
**ER**	Endoplasmic reticulum
**COX-2**	Cyclooxygenase-2
**AIF**	Apoptosis-inducing factor
**Bax**	Bcl-2-associated X Protein
**NSCLC**	Non-small-cell lung cancer
**PERK**	Protein kinase RNA-like endoplasmic reticulum kinase
**JAK2**	Janus kinase 2
**C4H**	Cinnamic acid 4-hydroxylase
**4CL**	4-Coumarate CoA ligase
**VEGF**	Vascular endothelial growth factor
**VEGFR**	Vascular endothelial growth factor receptor
**STAT3**	Signal transducer and activator of transcription
**NPs**	Nanoparticles
**CXCR7**	Chemokine receptor 7
**MMP**	Matrix metallopeptidase
**MAPK**	Mitogen-activated protein kinase
**JNK**	C-Jun N-terminal kinase
**PKC**	Protein kinase C
**TPA**	12-O-tetradecanoylphorbol-13-acetate
**FGF**	Fibroblast growth factor
**PDGF**	Platelet-derived growth factor
**HUVEC**	Human umbilical vein endothelial cell
**EPC**	Endothelial progenitor cell
**SDF-1α**	Stromal cell-derived factor 1α
**BM**	Bone marrow
**TME**	Tumor microenvironment
**HIF-1α**	Hypoxia-inducible factor 1α
**ATG**	Autophagy-related protein
**PARP**	Poly ADP ribose polymerase
**SQSTM1**	Sequestosome 1
**PTP**	Protein tyrosine phosphatase
**mTOR**	Mechanistic target of rapamycin
**SOD**	Superoxide dismutase
**NAC**	N-acetyl-L-cysteine
**HRCs**	Human primary renal epithelial cells
**MDR**	Multi-drug resistant
**CTSC**	Cathepsin C
**PBS**	Phosphate-buffered saline
**BRB**	Blood–retinal barrier
**MCL-1**	Myeloid cell leukemia 1
**Bcl-2**	B-cell lymphoma 2
**Bak**	Bcl-2 antagonist/killer
**PD-L1**	Programmed cell death-ligand 1
**Bcl-XL**	B-cell lymphoma-extra large
**PAL**	Phenylalanine ammonia-lyase
**U6PT**	Umbelliferone 6 prenyl transferase
**C2′H**	*p*-Coumaroyl CoA 2′-hydroxylase
